# ORAI1 channel gating and selectivity is differentially altered by natural mutations in the first or third transmembrane domain

**DOI:** 10.1113/JP277079

**Published:** 2018-11-28

**Authors:** M. Bulla, G. Gyimesi, J.H. Kim, R. Bhardwaj, M.A. Hediger, M. Frieden, N. Demaurex

**Affiliations:** ^1^ Department of Cell Physiology and Metabolism University of Geneva Geneva Switzerland; ^2^ Institute of Biochemistry and Molecular Medicine University of Bern Bern Switzerland; ^3^ Departments of Physiology and Global Medical Science Yonsei University Wonju College of Medicine Wonju Republic of Korea; ^4^ Mitohormesis Research Center Yonsei University Wonju College of Medicine Wonju Republic of Korea

**Keywords:** ORAI1, CRAC, STIM1, SOCE, TAM, calcium, GSK–7975A

## Abstract

**Key points:**

- Gain‐of‐function mutations in the highly selective Ca^2+^ channel ORAI1 cause tubular aggregate myopathy (TAM) characterized by muscular pain, weakness and cramping.
- TAM‐associated mutations in ORAI1 first and third transmembrane domain facilitate channel opening by STIM1, causing constitutive Ca^2+^ influx and increasing the currents evoked by Ca^2+^ store depletion.
- Mutation V107M additionally decreases the channel selectivity for Ca^2+^ ions and its inhibition by acidic pH, while mutation T184M does not alter the channel sensitivity to pH or to reactive oxygen species.
- The ORAI blocker GSK‐7975A prevents the constitutive activity of TAM‐associated channels and might be used in therapy for patients suffering from TAM.

**Abstract:**

Skeletal muscle differentiation relies on store‐operated Ca^2+^ entry (SOCE) mediated by STIM proteins linking the depletion of endoplasmic/sarcoplasmic reticulum Ca^2+^ stores to the activation of membrane Ca^2+^‐permeable ORAI channels. Gain‐of‐function mutations in STIM1 or ORAI1 isoforms cause tubular aggregate myopathy (TAM), a skeletal muscle disorder with muscular pain, weakness and cramping. Here, we characterize two overactive ORAI1 mutants from patients with TAM: V107M and T184M, located in the first and third transmembrane domain of the channel. When ectopically expressed in HEK‐293T cells or human primary myoblasts, the mutated channels increased basal and store‐operated Ca^2+^ entry. The constitutive activity of V107M, L138F, T184M and P245L mutants was prevented by low concentrations of GSK‐7975A while the G98S mutant was resistant to inhibition. Electrophysiological recordings confirmed ORAI1‐V107M constitutive activity and revealed larger STIM1‐gated V107M‐ and T184M‐mediated currents with conserved fast and slow Ca^2+^‐dependent inactivation. Mutation V107M altered the channel selectivity for Ca^2+^ ions and conferred resistance to acidic inhibition. Ca^2+^ imaging and molecular dynamics simulations showed a preserved sensitivity of T184M to the negative regulation by reactive oxygen species. Both mutants were able to mediate SOCE in *Stim1^−/−^/Stim2^−/−^* mouse embryonic fibroblasts expressing the binding‐deficient STIM1‐F394H mutant, indicating a higher sensitivity for STIM1‐mediated gating, with ORAI1‐T184M gain‐of‐function being strictly dependent on STIM1. These findings provide new insights into the permeation and regulatory properties of ORAI1 mutants that might translate into therapies against diseases with gain‐of‐function mutations in *ORAI1*.

## Key points

- Gain‐of‐function mutations in the highly selective Ca^2+^ channel ORAI1 cause tubular aggregate myopathy (TAM) characterized by muscular pain, weakness and cramping.
- TAM‐associated mutations in ORAI1 first and third transmembrane domain facilitate channel opening by STIM1, causing constitutive Ca^2+^ influx and increasing the currents evoked by Ca^2+^ store depletion.
- Mutation V107M additionally decreases the channel selectivity for Ca^2+^ ions and its inhibition by acidic pH, while mutation T184M does not alter the channel sensitivity to pH or to reactive oxygen species.
- The ORAI blocker GSK‐7975A prevents the constitutive activity of TAM‐associated channels and might be used in therapy for patients suffering from TAM.

## Introduction

The Ca^2+^ release‐activated Ca^2+^ (CRAC) channel ORAI1 is a highly Ca^2+^‐selective channel activated at the plasma membrane (PM) of eukaryotic cells by the depletion of endoplasmic reticulum (ER)/sarcoplasmic reticulum (SR) Ca^2+^ stores, a process termed store‐operated Ca^2+^ entry (SOCE) (Putney, 1986, 1990; Soboloff *et al*. 2012) that regulates various cellular functions such as gene expression, cell motility and muscle contraction (Berridge *et al*. 2003; Clapham, 2007; Stiber *et al*. 2008). The ER Ca^2+^ sensor STIM1 translocates to the PM to bind and gate ORAI channels following store depletion (Liou *et al*. 2005; Roos *et al*. 2005; Zhang *et al*. 2005), and mutations in *STIM1* or *ORAI1* genes lead to severe immunological or muscular diseases (Feske, 2009; Bohm *et al*. 2013; Hedberg *et al*. 2014; Misceo *et al*. 2014; Morin *et al*. 2014; Nesin *et al*. 2014; Endo *et al*. 2015; Walter *et al*. 2015; Bohm *et al*. 2017).

Following the identification of ORAI1 and STIM1 as the molecules mediating SOCE (Liou *et al*. 2005; Roos *et al*. 2005; Zhang *et al*. 2005; Feske *et al*. 2006; Zhang *et al*. 2006; Vig *et al*. 2006b), structural and mutagenesis studies unravelled key residues involved in the trapping and gating of ORAI1 by STIM1 and in the modulation of channel activity by environmental factors. The crystal structure of the *Drosophila melanogaster* Orai1 revealed that the functional channel has a hexameric structure (Hou *et al*. 2012), although a tetrameric conformation was proposed for human ORAI1 (Thompson & Shuttleworth, 2013; Cai *et al*. 2016; Yen *et al*. 2016). The structural data indicate that ORAI1 transmembrane (TM) domains assemble as concentric rings, with TM1 forming the channel pore (Zhou *et al*. 2010; Hou *et al*. 2012) surrounded by TM2–TM3, which provide stability to the structure (Amcheslavsky *et al*. 2015), and the TM4 outer ring transducing the gating signal generated by the binding of STIM1 to the ORAI1 C‐terminus (Calloway *et al*. 2010; Amcheslavsky *et al*. 2015). Mutagenesis studies revealed that TM1 and the extracellular loop between TM1 and TM2 hold negatively charged residues (E106, D110, D112, D114) conferring to the channel its characteristic high Ca^2+^ selectivity (Prakriya *et al*. 2006; Vig *et al*. 2006b; McNally *et al*. 2009; Frischauf *et al*. 2015), and that a glutamic acid residue in TM3 (E190) senses external pH variations (Beck *et al*. 2014; Tsujikawa *et al*. 2015). Three cysteines in TM2 and TM3 (C126, C143, C195) act as oxidant sensors (Bogeski *et al*. 2010; Alansary *et al*. 2016). Natural substitutions of TM1 residues facing the channel pore alter channel conductance and selectivity, but mutations in TM2, TM3 or TM4 also alter channel conductance (Nesin *et al*. 2014; Endo *et al*. 2015; Bohm *et al*. 2017), suggesting that the gating signal is relayed along non‐pore‐lining residues to control ORAI1 channel opening.

The initiation of skeletal muscle contraction relies on the conformational change of voltage‐gated Ca^2+^ channels located on invaginations of the PM (T‐tubules), induced by membrane depolarization. This change is transmitted to the ryanodine receptor localized at the SR membrane and allows Ca^2+^ release from the stores, a process known as excitation–contraction coupling (Block *et al*. 1988; Launikonis *et al*. 2003; Launikonis & Rios, 2007). As the process of excitation–contraction can occur in the absence of external Ca^2+^, the role of SOCE in this tissue has been overlooked. The situation changed after the report of SOCE in muscle cells (Kurebayashi & Ogawa, 2001), the clarification of its role in maintaining cytosolic Ca^2+^ levels during contraction (Koenig *et al*. 2018), and the discovery of the muscular pathologies associated with STIM1 and ORAI1 mutations (Lacruz & Feske, 2015). Gain‐of‐function mutations in *STIM1* and *ORAI1* genes are causally associated with tubular aggregate myopathy (TAM), a genetic disorder (MIM no. 160565) affecting skeletal muscle, characterized by muscle contractures, weakness and pain exacerbated by exercise (Salviati *et al*. 1985; Bohm *et al*. 2014). The disorder results from a homeostatic imbalance leading to Ca^2+^ overload, and muscle biopsies typically display accumulation of sarcoplasmic reticulum aggregates, the diagnostic signature of TAM. Serum creatine kinase (CK) levels are systematically elevated and illustrate muscle damage. The severity of TAM symptoms varies from one patient to another, and they are combined with miosis, ichthyosis, thrombocytopenia, asplenia, dyslexia and short stature in Stormorken syndrome (Stormorken *et al*. 1985). The progression of the disease is difficult to control as no cure is currently available. In this study, we characterize two *ORAI1* mutations associated with TAM and establish their susceptibility to SOCE inhibitors. To gain insight into the molecular mechanism of channel opening, we chose two mutations with distinct positions and clinical phenotype: V107M, located in TM1, next to the Ca^2+^ selectivity filter E106, causing severe muscular and extramuscular defects, and T184M, located in TM3 and leading to an asymptomatic hyperCKemia (Bohm *et al*. 2017). Both the V107M and T184M channels are constitutively active and mediate increased SOCE when expressed in HEK‐293T cells, as inferred from cytosolic Ca^2+^ recordings (Bohm *et al*. 2017).

The electrophysiological signature of the ORAI1 channel is the prototypical CRAC current (*I*_CRAC_), an inwardly rectifying, highly Ca^2+^‐selective current with a very positive reversal potential (*E*_rev_), and Ca^2+^‐dependent current inactivation occurring on a millisecond (fast) and minute (slow) time scale (Hoth & Penner, 1992; Zweifach & Lewis, 1995*a*,*b*). In this study, we investigated the electrophysiological features of V107M and T184M mutant variants and show that these two *ORAI1* mutations increase channel conductance without affecting fast and slow Ca^2+^‐dependent inactivation. The mutation at position 107 additionally alters the channel Ca^2+^ selectivity and its sensitivity to external pH and to STIM1‐mediated gating, whereas the main effect of the T184M mutation is to increase ORAI1 susceptibility to gating by the binding‐deficient STIM1‐F394H. We also validated the SOCE inhibitor GSK‐7975A as a potential drug for patients suffering from diseases caused by gain‐of‐function mutations such as TAM.

## Methods

### Plasmids

The ORAI1‐yellow fluorescent protein (YFP) construct was purchased from Addgene (Cambridge, MA, USA; plasmid no. 19756). Site‐directed mutagenesis using the Pfu Turbo DNA polymerase from Agilent Technologies (Santa Clara, CA, USA; 600250) was used to introduce TAM point mutations (c.319G>A and c.551C>T). Forward (fwd) and complementary reverse mutagenesis primers were as follows: 5′‐GGC AAT GGT GGA GAT GCA GCT GGA CGC TGA C‐3′ (fwd, V107M), 5′‐CTC CAC CGT CAT CGG CAT GCT GCT CTT CCT AGC TG‐3′ (fwd, T184M), 5′‐CTC GAC CAC CAT CAT GGT GCT CTT CGG CCT GAT CTT TAT CG‐3′ (fwd P245L) and 5′‐CTG ACC GAC AGT TCC AGG AGG ACA ACG AGG ACG CGG AGT TTG CCC GCT TAC AGG‐3′ (fwd L273D‐L276D). They were synthetized by Microsynth (Balgach, Switzerland). The mCherry–ORAI1 constructs were generated by *Apa*I and *Sac*I digestion of mutated ORAI1–YFP constructs, followed by ligation of the insert into mCherry–hORAI1 (a kind gift from Dr Matthias Seedorf, University of Heidelberg, Germany), or by site‐directed mutagenesis using the 5′‐CGG ACC TCG GCT CTG CTC TCC TCC TTC GCC ATG GTG GCA ATG G‐3′ (fwd G98S) and 5′‐GGC TGT GCA CCT GTT TGC GTT CAT GAT CAG CAC CTG CAT C‐3′ (fwd L138F). For SOCE and *I*_CRAC_ evaluation, mCherry–STIM1 (gift from Prof. Richard S. Lewis, Stanford University, USA) and cyan fluorescent protein (CFP)–STIM1 (Shen *et al*. 2011) were co‐transfected with ORAI1 wild‐type (WT) or mutated constructs. The TagRFP‐KDEL (Guido *et al*. 2015) plasmid was used as a negative control in Ca^2+^ experiments. The gating‐deficient STIM1‐F394H–mCherry was generated by site‐directed mutagenesis of STIM1–mCherry (a kind gift from Dr Matthias Seedorf, University of Heidelberg, Germany) using the 5′‐CAC ACT CTT TGG CAC CCA CCA CGT GGC CCA CAG C‐3′ (fwd F394H). When co‐transfected, a 3:1 ratio by mass of STIM1:ORAI1 was used. All plasmids coded for human proteins.

### Cell culture

Human embryonic kidney (HEK‐293T) cells were obtained from ATCC (CRL‐11268, Manassas, VA, USA) and *Stim1^−/−^/Stim2^−/−^* mouse embryonic fibroblasts (DKO) were engineered by the group of Masatsugu Oh‐Hora (Tokyo Medical and Dental University, Japan). Cells were maintained at 37°C with 5% CO_2_, in DMEM (cat. no. 31966‐021 (HEK‐293T) and 15140‐122 (*Stim1^−/−^/Stim2^−/−^* MEF) from Thermo Fisher Scientific, Waltham, MA, USA), completed with 10% fetal bovine serum, 5 units ml^−1^ penicillin and 5 μg ml^−1^ streptomycin (cat. nos. 10270‐106 and 15140‐122, Thermo Fisher Scientific). Human primary myoblasts were obtained and cultured as previously described (Laumonier *et al*. 2017). The different ORAI1 constructs were electroporated with the Amaxa Nucleofector II device (Lonza, Basel, Switzerland).

All work on human subjects was carried out in accordance with the guideline and regulations of the Swiss Regulatory Health Authorities and approved by the University Hospital of Geneva Research Committee on the use of humans as experimental subjects (Protocol CER no. 12‐259).

For Ca^2+^ microscopy and patch‐clamp experiments, cells were seeded on glass coverslips before electroporation (myoblasts) or transfection (HEK‐293T, DKO cells) with 5 μl Lipofectamine® 2000 (cat. no. 11668‐019; Thermo Fisher Scientific) and a total of 2 μg STIM1:ORAI1 DNA. When STIM1 was co‐expressed with TAM mutated channels, the transfection mix was removed after 3–4 h, and cells were kept in a low‐Ca^2+^‐containing medium (addition of 1.7 mm EGTA to the complete medium and pH correction with NaOH) to prevent Ca^2+^ toxicity. All experiments were performed within 24–48 h post‐transfection. For fluorescence imaging plate reader (FLIPR) experiments, HEK‐293T cells were seeded at 30 000 cells/well density onto Corning® 96‐well black polystyrene clear bottom microplates (cat. no. CLS3603; Sigma‐Aldrich, St Louis, MO, USA) coated with 100 μg ml^−1^ poly‐d‐lysine (cat. no. P6407; Sigma‐Aldrich) and were transiently transfected with 100 ng/well of the WT or mutated forms of mCherry–ORAI1 using 0.3 μl Lipofectamine 2000/well. Cells were maintained at 37°C in a 0.2 mm Ca^2+^‐containing medium for 20 h before performing the experiment.

### Ca^2+^ measurements

HEK‐293T cells, DKO fibroblasts or primary human myoblasts were loaded with 4 μm Fura‐2 AM and 1 μm Pluronic acid F‐127 (F‐1201 and P‐3000MP, Thermo Fisher Scientific). They were kept in the dark at room temperature for 30 min before being washed. Fluorescence was recorded with a Nikon eclipse Ti microscope (Nikon Instruments, Zurich, Switzerland) equipped with a Lambda XL lamp (Sutter Instrument, Novato, CA, USA) and a 16‐bit CMOS cooling camera (pco.Edge sCMOS, Visitron Systems, Puchheim, Germany). The filter wheel (Ludl Electronic Products, Hawthorne, NY, USA) allowed a rapid change of the excitation filters (ET340x and ET380x, Chroma) and Fura‐2 ratiometric fluorescence was collected through the T400lp–ET510/80m beam splitter–emission cube from Chroma. Cells were exposed for 200 and 100 ms to 340 and 380 nm light respectively, and acquisitions were obtained every 2–5 s using VisiView software (Visitron Systems). ER store depletion was elicited by treating cells with 1 μm thapsigargin (Tg; cat. no. T9033; Sigma‐Aldrich) in a Ca^2+^‐free solution (1 mm EGTA), and SOCE was measured as the slope of the response after Ca^2+^ readmission. To assess the basal activity of ORAI1, 500 μm MnCl_2_ was added to the Ca^2+^‐containing recording solution, and Fura‐2 quench rate was evaluated at the dye's isosbestic point (360 nm). Experiments were performed at room temperature. The recording solutions contained 5 mm KCl, 140 mm NaCl, 1 mm MgCl_2_, 10 mm HEPES, 10 mm d‐glucose and 0.5, 1 or 2 mm CaCl_2_ (pH 7.4). For the Ca^2+^‐free solution, CaCl_2_ was replaced by 1 mm EGTA. One millimolar hydrogen peroxide (H_2_O_2_; 516 813, Sigma‐Aldrich) was added in all recording solutions to assess reactive oxygen species sensitivity of ORAI1‐T184M. For inhibition studies of TAM mutants, 10 μm GSK‐7975A (cat. no. AOB4124‐1; Aobious, Gloucester, MA, USA) or 0.1% DMSO (control, 0 μm GSK‐7975A) was added to recording solutions. ORAI1–YFP positive cells were selected with the ET500/20x–T515lp–ET535/30m cube (Chroma). In co‐expression experiments, only ORAI1–STIM1 double positive cells were followed, where red fluorescence was collected through the ET572/35x–69002bs–ET630/75m cube (Chroma), and only cells expressing comparable fluorescence levels of ORAI1 and similar STIM1:ORAI1 fluorescence ratios were considered for statistical evaluation. For FLIPR experiments, the growth medium of the HEK‐293T cells was removed 20 h after transfection and the cells were loaded with 50 μl of the Calcium 5 dye (FLIPR® Calcium 5 assay kit, R8186, Molecular Devices, Sunnyvale, CA, USA) prepared in modified Krebs buffer containing 0.2 mm CaCl_2_, 140 mm NaCl, 4.8 mm KCl, 1 mm MgCl_2_, 10 mm d‐glucose and 10 mm HEPES (pH 7.4). Cells were incubated in the loading buffer at 37°C for 30 min and were then pre‐incubated with 50 μl of various doses of GSK‐7975A in 0.2 mm CaCl_2_ containing Krebs buffer for another 30 min. The cells were excited using a 470–495 nm LED module of the FLIPR, and the emitted fluorescence signal was filtered with a 515–575 nm emission filter. After recording a baseline for 50 s, 50 μl of 5.6 mm CaCl_2_‐containing Krebs buffer together with GSK‐7975A was administered to the cells, resulting in 2 mm final concentration of Ca^2+^ and maintaining the same concentration of GSK‐7975A. The changes in fluorescence intensity were measured for first 90 s after CaCl_2_ administration with an acquisition rate of 2 Hz and for further 240 s with 0.5 Hz. The fluorescence signals were analysed using the FLIPR Tetra software, ScreenWorks 3.1.1.8 (Molecular Devices). To calculate the constitutive Ca^2+^ entry dedicated to each mutant, the area under the curve (AUC) of the WT ORAI1 was subtracted from each ORAI1 mutant response. Normalized data were used to extract the GSK‐7975A half‐inhibitory concentration (IC_50_) for each ORAI1 variant.

### Electrophysiology

Transiently transfected HEK‐293T cells were trypsinized, plated on poly‐l‐lysine‐coated (cat. no. P1274; Sigma‐Aldrich) coverslips and incubated for at least 1 h at 37°C to allow the attachment of separated cells. Only cells expressing comparable fluorescence levels of ORAI1–YFP and similar CFP–STIM1:ORAI1–YFP fluorescence ratios were considered. The experiments were performed at room temperature, in the whole‐cell configuration. Pipettes were pulled from 1.5 mm thin‐wall glass capillaries (World Precision Instruments, Hitchin, UK) using a vertical PC‐10 Narishige puller to obtain a resistance of around 2 mΩ. Currents were recorded with pCLAMP 10.7 software (Molecular Devices), using an Axopatch 200B amplifier (Molecular Devices) with a low‐pass filtering at 1 kHz, and digitized with the Axon Digidata 1550A at 1 ms. Voltage ramps of 400 ms from −120 to +70 mV were applied to cells, from a holding potential of 0 mV. Currents at −110 mV were considered to report the maximal current density and were corrected by the cell capacitance (pA pF^−1^). All currents were corrected for leak by subtracting the residual current after blocking with 10 μm GdCl_3_. The standard 10 mm Ca^2+^ recording solution contained 130 mm NaCl, 5 mm CsCl, 5 mm KCl, 1 mm MgCl_2_, 10 mm CaCl_2_ and 10 mm HEPES (310–320 mOsm, pH 7.4 corrected with NaOH). The divalent free (DVF) solution was 145 mm NaCl, 5 mm EGTA, 2 mm EDTA and 20 mm HEPES. The intracellular pipette solution contained 8 mm NaCl, 130 mm caesium methansulfonate, 3.5 mm MgCl_2_, 10 mm EGTA, 2 μm Tg and 10 mm HEPES (280–290 mOsm, pH 7.2 corrected with CsOH). To measure slow Ca^2+^‐dependent inactivation (SCDI), intracellular EGTA was decreased to 1.2 mm. After establishment of the whole‐cell configuration, cells were kept in a nominal Ca^2+^‐free (NCF) bath for 2–3 min to allow store depletion before exposure to 10 mm Ca^2+^. NCF solution contained 140 mm NaCl, 10 mm CsCl, 5 mm KCl, 3 mm MgCl_2_ and 10 mm HEPES (pH 7.4). SCDI was expressed as the fraction of current remaining at steady state compared to the maximal current amplitude. Fast Ca^2+^‐dependent inactivation (FCDI) was recorded by applying 200 ms hyperpolarizing voltage pulses of −120, −100, −80 and −60 mV, from a holding potential of 0 mV, with 1 kHz filtration and 5 kHz sampling. The pipette solution contained 10 mm EGTA. FCDI was recorded after full development and stabilization of the current and expressed as the fraction of current remaining 195 ms after the current peak. The peak was determined 1.5 ms after the start of the pulse to minimize the contribution of uncompensated capacitance. To assess the channel selectivity for Ca^2+^ ions, NaCl in the external solution was replaced by 135 mm *N*‐methyl‐d‐glucamine (NMDG; pH 7.4 corrected with HCl) and the fraction of current remaining in the NMDG solution was reported. The current reversal potential (*E*_rev_) in the presence or absence of STIM1 co‐expression was calculated using the third‐order polynomial equation *y* = *B*_0_ *+ B*_1_ *x + B*_2_ *x^2^ + B*_3_ *x^3^*. To evaluate the impact of an acidic environment on the current amplitude, cells were exposed to media of decreasing pH as HEPES‐ and MES‐buffered solutions of pH 7.4 and 7.0 and 6.6, 6.2 and 5.8, respectively. Normalization to the maximal current at pH 7.4 allowed the estimation of the fractional block at each pH step.

### Human ORAI1 model generation and molecular dynamics simulations

Sequence alignment between the sequences of the crystallized *D. melanogaster* construct (PDB ID: 4HKR) and human ORAI1 (UniProt ID: Q96D31) was created using ClustalW2 default settings (Larkin *et al*. 2007). Sixty‐three per cent of residues identified in the crystallized protein were identical to their corresponding residues in human ORAI1. The human sequence was threaded on the X‐ray coordinates, downloaded from the Orientations of Proteins in Membranes (OPM) database (Lomize *et al*. 2006), using the Rosetta 2016.20.58704 ‘fixbb’ program (Kuhlman *et al*. 2003), followed by side‐chain repacking. Residues 72–286 were modelled altogether, loops (residues 109–118, 148–163, 206–234) were added using the ‘loopmodel’ program in Rosetta, with remodelling using the cyclic coordinates descent (Canutescu & Dunbrack, 2003), and refinement using the kinematic closure algorithm (Mandell *et al*. 2009). Fragments for modelling were created using the Robetta server (http://robetta.bakerlab.org/). The smoothed all‐atom membrane scoring function was applied (Barth *et al*. 2007), and membrane‐spanning regions were defined according to the OPM database. The model with the best score was taken as the final model. Later, side‐chain conformations of E106 were adjusted using Pymol 2.1.0 (https://pymol.org/2/) to reflect the original conformation in the 4HKR PDB structure. Our model of human ORAI1 was embedded in a palmitoyl‐oleoyl‐phosphatidylcholine (POPC) bilayer, water, neutralizing ions (Cl^−^) and 150 mm NaCl using CHARMM‐GUI (Jo *et al*. 2008; Jo *et al*. 2014; Wu *et al*. 2014; Lee *et al*. 2016) with default options. The protein chain termini were capped by acetylation/methylation, and residue E190 was protonated. A single Ca^2+^ ion was placed in the location of the Ba^2+^ ion observed in the crystals. Simulations were performed using the CHARMM36 force‐field (Klauda *et al*. 2010; Best *et al*. 2012) in an NPγT ensemble at zero surface tension and 310 K temperature using AMBER16 (Case *et al*. 2016) on UBELIX (http://www.id.unibe.ch/hpc), the HPC cluster at the University of Bern. The analyses of the trajectories were performed after fitting all frames onto the initial structure using the C_α_ atoms of residues 69–102 (TM1). For the water and ion density plots, all water oxygen atoms, as well as Na^+^ and Cl^−^ ions within a 10 Å radius around the pore axis were collected. The axial angle of residue F99 was calculated as described in Yeung *et al*. (2018). Analyses were performed using MDAnalysis 0.18.0 (Michaud‐Agrawal *et al*. 2011; Gowers *et al*. 2016) and also used the ‘sasa’ tool included with GROMACS 2016 (Hess *et al*. 2008; Abraham *et al*. 2015). Structural figures were prepared using PyMOL 1.8.2.1. Graphs in Fig. 8 were generated using the seaborn 0.9.0 (https://seaborn.pydata.org/) distplot and catplot functions.

### Analysis software and statistics

Ca^2+^ experiments recorded with Visiview (Visitron Systems) were analysed with Fiji (ImageJ). Clampfit 10.7 (Molecular Devices) was used to extract currents from electrophysiology recordings. Statistical evaluation of the data was performed using Prism 7.02 (GraphPad Software, La Jolla, CA, USA); the sample size of each data set and the statistical test used are indicated in the figure legends. Data are expressed as mean ± 95% confidence interval (CI). The D'Agostino–Pearson test was used to assess the normality of the data distribution, and non‐parametric statistical tests were used for samples of small sizes and for data following a non‐Gaussian distribution. *P* values are labelled with asterisks: ^*^ *P* ≤ 0.05, ^**^ *P* ≤ 0.01, ^***^ *P* ≤ 0.001 and ^****^ *P* ≤ 0.0001.

### Data availability

All data supporting the findings of this study are available within the article. The primary and secondary data generated in the course of this project are available from the corresponding author upon request.

## Results

### The constitutive activity of TAM ORAI1 channels is sensitive to GSK‐7975A

**Figure 1:**
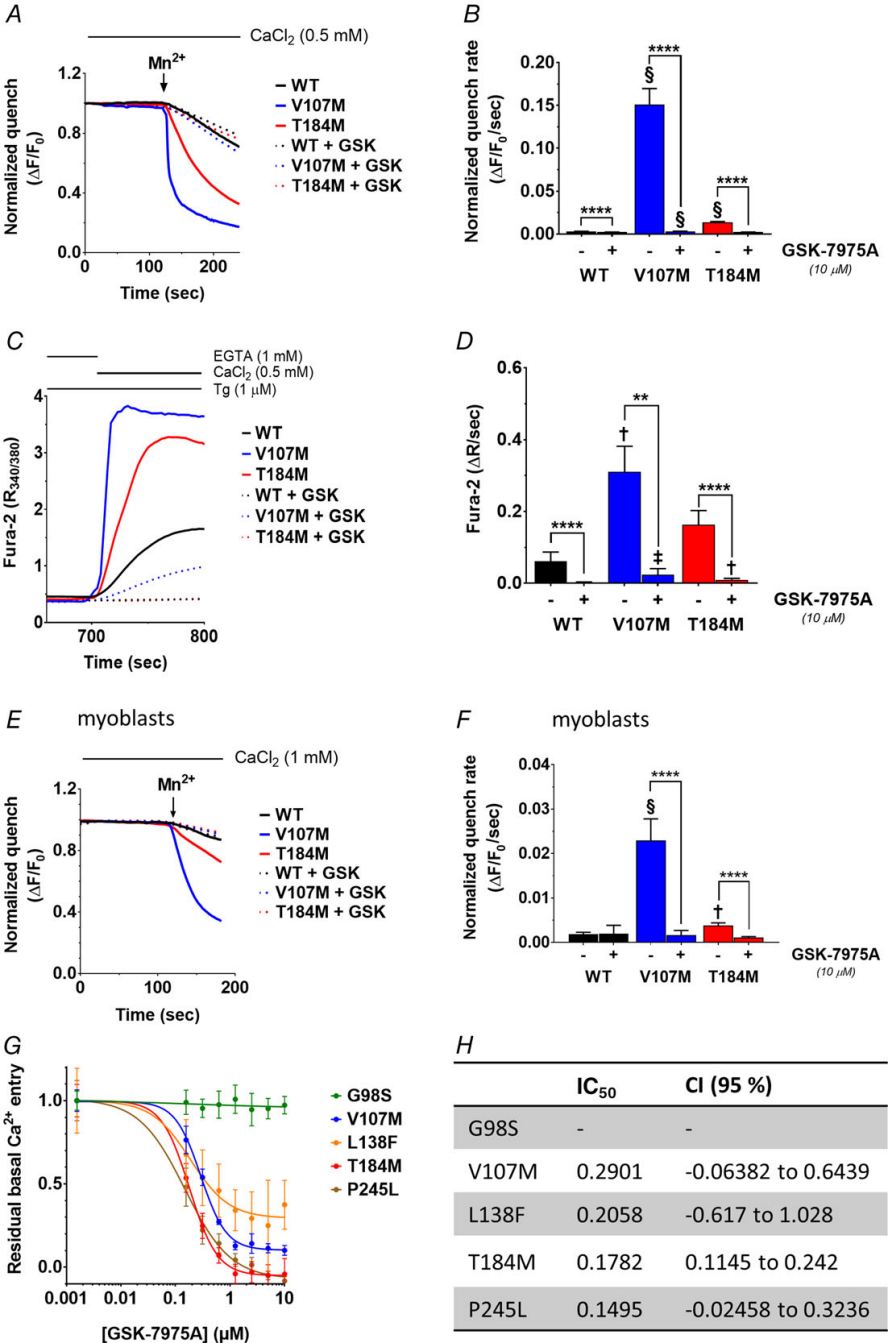
Inhibition of constitutively active TAM channels by GSK‐7975A *A*, mean recordings of Mn^2+^‐induced Fura‐2 fluorescence quench in store‐replete HEK‐293T cells transiently expressing ORAI1‐WT, ‐V107M or ‐T184M, treated or not with 10 μm GSK‐7975A. Mn^2+^ (500 μm) was added to the Ca^2+^ recording solution when indicated (arrow) and the quench rate assessed at the isosbestic point of Fura‐2 (360 nm). *B*, statistical evaluation of the quench rates. Data are means ± 95% CI of 96–231 cells from 2 independent experiments. *C*, representative Fura‐2 ratio fluorescence responses evoked by readmitting Ca^2+^ to thapsigargin (Tg)‐treated HEK‐293T cells co‐expressing STIM1 and ORAI1‐WT, ‐V107M or ‐T184M, treated or not with 10 μm GSK‐7975A. *D*, statistical evaluation of the Ca^2+^ influx rates, assessed by measuring the slope of the response in *C*. Data are means ± 95% CI of 14–54 cells from 3 independent experiments. *E*, mean recordings of Mn^2+^‐induced Fura‐2 quench in store‐replete human primary myoblasts transiently expressing ORAI1‐WT, ‐V107M or ‐T184M, treated or not with 10 μm GSK‐7975A. *F*, statistical evaluations of the quench rates. Data are means ± 95% CI of 21–54 cells from 3 independent experiments. In *B*, *D* and *F*, the two‐tailed Kruskal–Wallis test with Dunn's correction was used to show statistical differences between treated and untreated groups (^****^ *P* ≤ 0.0001, ^**^ *P* ≤ 0.01) or between the WT and each of the mutated ORAI1 in treated or untreated conditions (§*P* ≤ 0.0001, ‡*P* ≤ 0.001, †*P* ≤ 0.01). *G*, GSK‐7975A dose–response curves of normalized constitutive Ca^2+^ entry in populations of HEK‐293T cells transfected with the TAM‐associated ORAI1‐G98S, ‐V107M, ‐L138F, ‐T184M and ‐P245L. Ca^2+^ levels were assessed with Calcium 5 in a plate reader, as described in Methods. *H*, half‐inhibitory concentration (IC_50_) of the constitutive Ca^2+^ entry through the mutated ORAI1 channels shown in *G*. Data are means ± 95% CI of 2–3 independent plates (12–18 wells).

We previously showed that ORAI1 channels carrying TAM‐associated mutations are constitutively active when expressed in HEK‐293T cells with replete stores (Bohm *et al*. 2017). Upon store depletion, they form clusters comparable to the wild‐type (WT) channel, but mediate increased SOCE. We therefore tested whether these overactive channels could be blocked by an ORAI1 inhibitor. We considered GSK‐7975A, based on its specificity for CRAC channels (ORAI1 and ORAI3), and on its effectiveness supported by models of acute pancreatitis (Derler *et al*. 2008; Gerasimenko *et al*. 2013). HEK‐293T cells overexpressing comparable levels of the WT or mutated TAM ORAI1 (V107M or T184M) were loaded with the Ca^2+^‐sensitive dye Fura‐2, and the basal activity of the channels was evaluated using the manganese (Mn^2+^) quench assay, in the presence or absence of GSK‐7975A (Fig. 1 *A*). Mn^2+^ permeates through open PM channels and quenches Fura‐2 fluorescence, enabling the isolation of the Ca^2+^ influx component from the activity of other Ca^2+^ pumps or exchangers by recording Fura‐2 quench rates at its isosbestic point (360 nm). In accordance with our previous observations (Bohm *et al*. 2017), Fura‐2 quench rates were 4–50 times larger in cells expressing TAM channels than WT channels, confirming their basal activity in this cellular model (Fig. 1 *B*). The addition of 10 μm GSK‐7975A efficiently blocked the basal Mn^2+^ ‘leak’ across both TAM channels.

We next depleted ER Ca^2+^ stores with thapsigargin (Tg) and evaluated SOCE in HEK‐293T cells co‐expressing ORAI1‐WT, ‐V107M or ‐T184M together with STIM1 (Fig. 1 *C* and *D*), at comparable protein ratio. Upon Ca^2+^ readmission, the rates of the evoked Fura‐2 ratio elevations were ∼2.5 and 5 times larger for ORAI1‐T184M and ‐V107M, respectively, compared to WT channels, confirming our earlier findings (Bohm *et al*. 2017). The addition of 10 μm GSK‐7975A efficiently reduced SOCE in all conditions.

We then tested the effects of GSK‐7975A in human primary myoblasts transiently expressing the ORAI1‐V107M and ‐T184M mutants. As observed in HEK‐293T cells, Mn^2+^‐induced Fura‐2 quench rates were 2–12 times larger in cells expressing ORAI1‐T184M and ‐V107M compared to WT, and were abolished by 10 μm GSK‐7975A (Fig. 1 *E* and *F*). These data indicate that the two ORAI1 TAM mutants mediate constitutive Ca^2+^ entry in skeletal muscle and that this constitutive activity is inhibited by GSK‐7975A.

To appraise the possible application of GSK‐7975A in therapeutics for TAM patients, we established the effects of this compound on three additional TAM‐associated ORAI1 channel mutants, using a high‐throughput fluorescence imaging plate reader (FLIPR). HEK‐293T cells transiently expressing the constitutively active G98S, V107M, L138F, T184M or P245L ORAI1 channels (Nesin *et al*. 2014; Endo *et al*. 2015; Bohm *et al*. 2017) were loaded with the Ca^2+^‐sensitive dye Calcium 5 and incubated with increasing GSK‐7975A concentrations, from a subminimal (0.0016 μm) to a maximal (10 μm) dose. Normalized areas under curve (AUC) after subtraction of the WT baseline response were used to determine the half‐inhibitory GSK‐7975A concentration (IC_50_) for each TAM variant (Fig. 1 *G* and *H*). The constitutive activity of four of the five TAM‐associated ORAI1 mutations (ORAI1‐V107M, ‐L138F, ‐T184M and ‐P245L) was efficiently blocked by submicromolar GSK‐7975A concentrations, while the pore‐mutated ORAI1‐G98S channel was resistant to inhibition (Fig. 1 *G* and *H*). These data indicate that GSK‐7975A blocks constitutive Ca^2+^ entry in myoblasts expressing TAM‐associated ORAI1 channels and suggest that this compound could provide therapeutic benefit in a broad subset of, but not all, TAM patients.

### Biophysical properties of TAM‐associated ORAI1 channels

**Figure 2:**
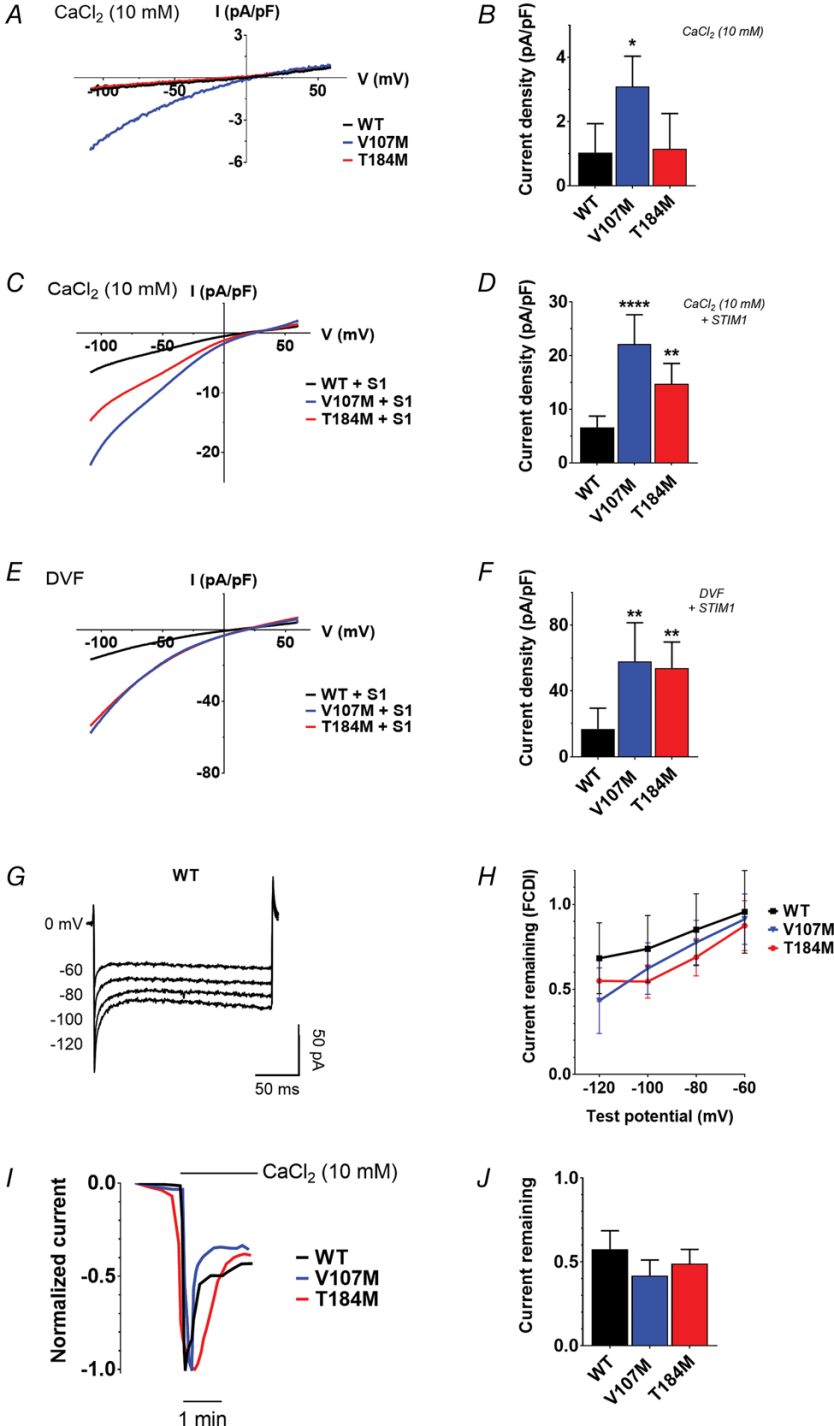
Electrophysiological recordings of TAM‐associated ORAI1 channels *A*, representative *I*_CRAC_ recordings in HEK‐293T cells expressing ORAI1‐WT, ‐V107M or ‐T184M without STIM1, treated with Tg and exposed to 10 mm Ca^2+^. Currents were evoked by voltage ramps from −120 to 70 mV. *B*, statistical evaluation of current densities in *A*, at −110 mV. Data are means ± 95% CI of 7 cells for each condition (two‐tailed Kruskal–Wallis test). *C*, mean *I*_CRAC_ recordings in 10 mm Ca^2+^ of cells co‐expressing WT or mutant ORAI channels together with STIM1 and treated with Tg. All currents are inwardly rectifying with a positive reversal potential (*E*_rev_). *D*, statistical evaluation of current densities at −110 mV in *C*. Data are means ± 95% CI of 14–16 cells (two‐tailed Kruskal–Wallis test). *E*, mean current recordings in divalent free (DVF) solution of cells expressing STIM1 and ORAI1 WT or mutant channels, treated with Tg. *F*, statistical evaluation of the current densities at −110 mV in *E*. Data are means ± 95% CI of 6–9 cells (two‐tailed Kruskal–Wallis test). *G*, illustrative recordings of *I*_CRAC_ fast Ca^2+^‐dependent inactivation (FCDI) in HEK‐293T cells co‐expressing STIM1 and ORAI1‐WT during voltage pulses of −120, −100, −80 and −60 mV in 10 mm Ca^2+^. The pipette solution contained 10 mm EGTA and 2 μm Tg. *H*, fraction of current remaining 195 ms after each hyperpolarizing voltage step (mean ± 95% CI of 7–11 cells). Two‐way ANOVA and Dunnett's comparison tests were used to compare WT and TAM‐variant channels at each voltage step. *I*, illustrative recordings of *I*_CRAC_ slow Ca^2+^‐dependent inactivation in cells co‐expressing STIM1 and ORAI1‐WT, ‐V107M or ‐T184M. Cells were kept in nominal Ca^2+^‐free solution before acute exposure to 10 mm Ca^2+^. The internal solution contained 1.2 mm EGTA and 2 μm Tg. *J*, statistical evaluation of SCDI as the fraction of current remaining after stabilization of the current in 10 mm Ca^2+^ (mean ± SEM of 6–11 cells, two‐tailed Kruskal–Wallis test).

To gain insight into the gating and permeation properties of ORAI1 channels carrying TAM mutations, we recorded *I*_CRAC_ in HEK‐293T cells overexpressing WT, V107M or T184M channels. Pipette solutions contained 10 mm EGTA and 2 μm Tg to deplete ER Ca^2+^ stores, and the external solution contained 10 mm Ca^2+^. Voltage ramps (−120 to +70 mV) were applied at 5 s intervals to record *I*_CRAC_ development, and 10 μm Gd^3+^ was added at the end of the recordings to estimate the leak subtraction and determine the current density at −110 mV. In the absence of STIM1 co‐expression, a slightly inwardly rectifying current of small amplitude developed in cells expressing ORAI1‐V107M, which was not apparent in cells expressing ORAI1‐WT or ‐T184M, illustrating the STIM1‐independent constitutive activity of ORAI1‐V107M (Fig. 2 *A* and *B*). When STIM1 was co‐expressed together with ORAI1, both TAM mutated channels mediated significantly larger *I*_CRAC_ than WT channels (Fig. 2 *C* and *D*), an effect that persisted in a divalent free (DVF) solution, with V107M and T184M mediating ∼3 times larger currents than the WT current (Fig. 2 *E* and *F*). Interestingly, the V107M‐mediated currents were larger than the T184M currents when recorded in 10 mm Ca^2+^, but were of similar amplitude when recorded in DVF (compare Fig. 2 *C* and *D* with Fig. 2 *E* and *F*).

We next tested the fast (milliseconds) Ca^2+^‐dependent inactivation (FCDI) of the TAM mutated channels reflecting the negative feedback exerted by Ca^2+^ ions on the ORAI1 pore (Zweifach & Lewis, 1995*a*; Yamashita *et al*. 2007; Derler *et al*. 2009; Lee *et al*. 2009; Srikanth *et al*. 2010) and the slow (minutes) Ca^2+^ inactivation component (SCDI) reportedly mediated by calmodulin and SOCE‐associated regulatory factor (SARAF) (Zweifach & Lewis, 1995*b*; Parekh, 1998; Mullins *et al*. 2009; Palty *et al*. 2012; Jha *et al*. 2013). FCDI, assessed by brief negative voltage steps, was not significantly different in V107M, T184M and WT channels when currents of similar amplitudes were compared (Fig. 2 *G* and *H*). Similarly, SCDI, assessed by applying 10 mm Ca^2+^ after maximal SOCE activation to cells perfused with low EGTA concentrations (1.2 mm), was comparable in TAM and WT channels (Fig. 2 *I* and *J*).

### ORAI1‐V107M is permeable to sodium

**Figure 3:**
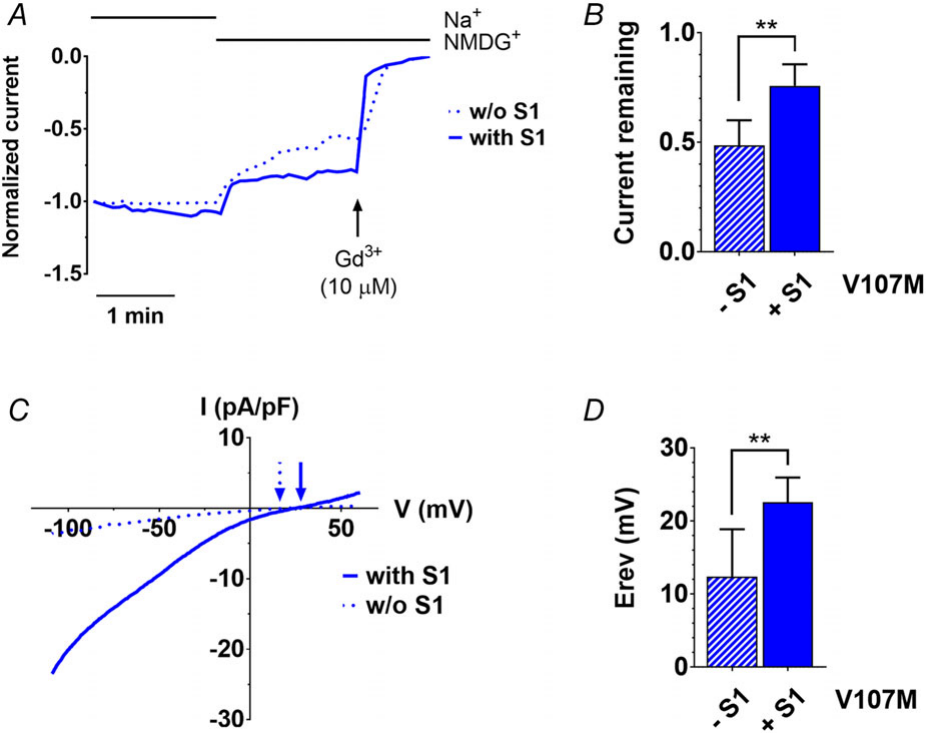
ORAI1‐V107M is permeable to sodium *A*, representative normalized current recordings of HEK‐293T cells expressing ORAI1‐V107M, with or without STIM1. When indicated, Na^+^ was replaced by the impermeant ion NMDG^+^ to assess the contribution of sodium ions to the inward currents recorded at −110 mV. *B*, fractional current remaining after Na^+^ removal after leak subtraction (Gd^3+^) in recordings illustrated in *A*. *C*, representative *I–V* curves of HEK‐293T cells expressing ORAI1‐V107M, with or without STIM1, recorded in a Ca^2+^‐ (10 mm) and Na^+^‐containing medium. Arrows indicate the corresponding reversal potential (*E*_rev_). *D*, statistical evaluation of *E*_rev_ recorded in the presence or absence of STIM1, as illustrated in *C*. Data are means ± 95% CI of 7–10 cells in *B* and *D* (two‐tailed Mann–Whitney test). [Color figure can be viewed at wileyonlinelibrary.com]

STIM1 tunes the Ca^2+^ selectivity of ORAI channels and confers high Ca^2+^ selectivity to the ORAI1‐V102C channel, which exhibits significant sodium (Na^+^) permeation and a left‐shifted reversal potential (*E*_rev_) at low STIM1 levels (McNally *et al*. 2012). Because the V107 residue is adjacent to the glutamic acid (E106) forming the Ca^2+^ selectivity filter of ORAI1 (Prakriya *et al*. 2006; Vig *et al*. 2006a; McNally *et al*. 2009), we tested whether the methionine substitution at position 107 impacts channel selectivity. For this, we recorded ORAI1‐V107M currents in a physiological saline buffer and replaced Na^+^ by the non‐permeant NMDG^+^ ion. The currents decreased by 20% upon Na^+^ removal when V107M was co‐expressed with STIM1, indicating that the mutated channel is permeable to Na^+^ ions. A more pronounced decrease (50%) was observed when V107M was expressed without STIM1, indicating that the constitutively active channel is even more permeable to Na^+^ ions (Fig. 3 *A* and *B*). Accordingly, *E*_rev_ was close to 10 mV when ORAI1‐V107M was expressed without STIM1, and shifted to more positive voltages when STIM1 was co‐expressed (Fig. 3 *C* and *D*). Consistent with the reported role of STIM1 in tuning the Ca^2+^ selectivity of ORAI1 channels, these data indicate that ORAI1‐V107M is permeable to Na^+^ and regains partial Ca^2+^ selectivity at high STIM1 levels.

### ORAI1‐V107M is resistant to acidic pH block

**Figure 4:**
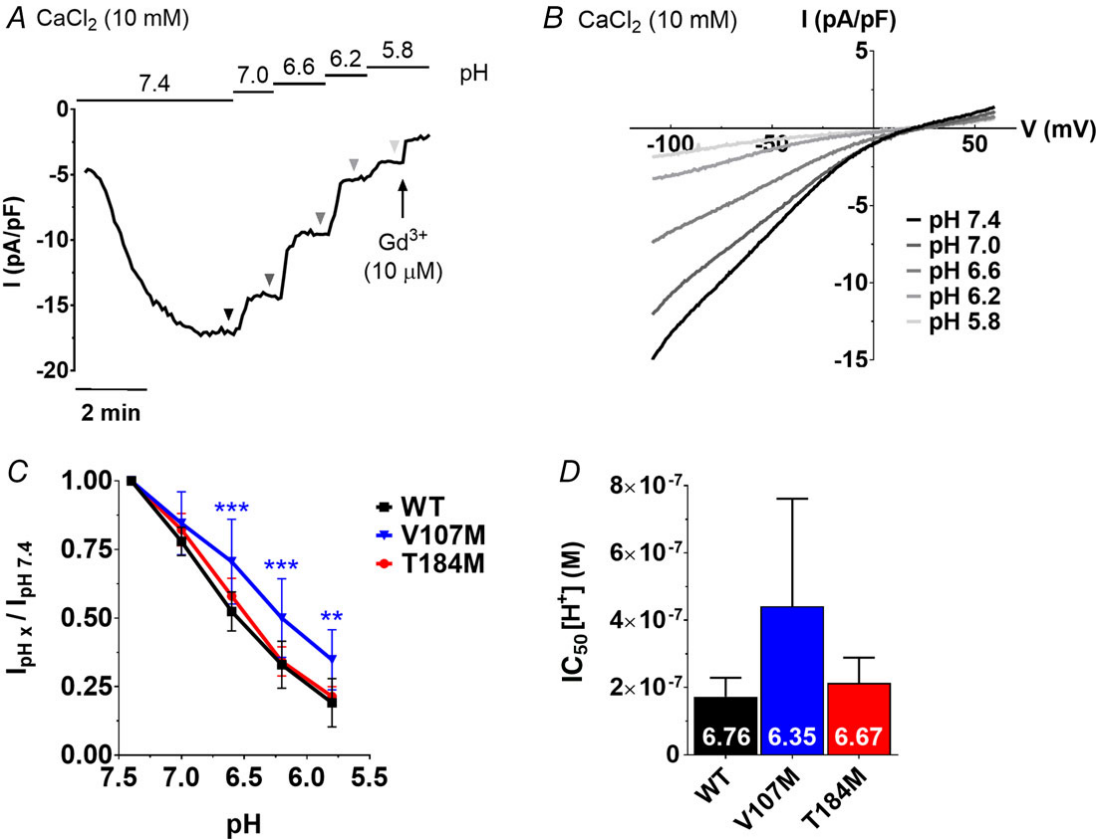
ORAI1‐V107M is resistant to acidic inhibition *A*, representative trace of a HEK‐293T cell co‐expressing STIM1 and ORAI1‐WT, exposed to Ca^2+^ solutions of decreasing pH. *B*, ramp currents recorded at each pH step are indicated by triangles in *A*. *C*, fractional current recorded at different acidic pH, in cells expressing the WT or mutated channels. *D*, half‐inhibitory concentration (IC_50_) of WT and TAM currents by protons (H^+^). The corresponding pH are indicated in white on each bar. Data are means ± 95% CI of 6–7 cells. Two‐way ANOVA and Dunnett's comparison test were used to evaluate statistical differences between the mutated and the WT channels in *C*. Statistical significance was not reached in *D* with the use of the low power Kruskal–Wallis test (Dunn's correction).

The V107 residue is located close to a cluster of negatively charged residues (E106, D110, D112, D114) involved in the pH modulation of the ORAI1 channel (Beck *et al*. 2014) while the T184 residue is close to a glutamic acid residue in the TM3 of ORAI1 (E190) reported to mediate external pH sensing (Tsujikawa *et al*. 2015). Mutations at positions 106, 110, 112 or 190 alter the external pH dependency of *I*_CRAC_, which is normally potentiated at alkaline pH and inhibited at acidic pH. We therefore tested whether the two TAM mutations attenuate ORAI1 pH sensitivity. HEK‐293T cells were co‐transfected with STIM1 and ORAI1‐WT or TAM channels and the external pH was acutely decreased after *I*_CRAC_ had reached steady state. As previously reported, the amplitude of *I*_CRAC_ mediated by WT ORAI1 channels decreased as the external pH acidified from 7.4 to 5.8, the residual currents at pH 5.8 averaging 20% of the currents recorded at physiological pH (Fig. 4 *A–C*). An identical pH sensitivity was observed for currents mediated by ORAI1‐T184M, while ORAI1‐V107M‐mediated currents were significantly larger at acidic pH (Fig. 4 *C*). The fractional inhibition of V107M currents was reduced and half‐maximal inhibition was achieved at a pH that was ∼0.4 units more acidic for the V107M channel than for WT or T184M channels (Fig. 4 *D*). These data indicate that the V107M mutation in TM1, but not the T184M mutation in TM3, alters the channel sensitivity to external pH.

### ORAI1‐T184M is sensitive to H_2_O_2_ inhibition

**Figure 5:**
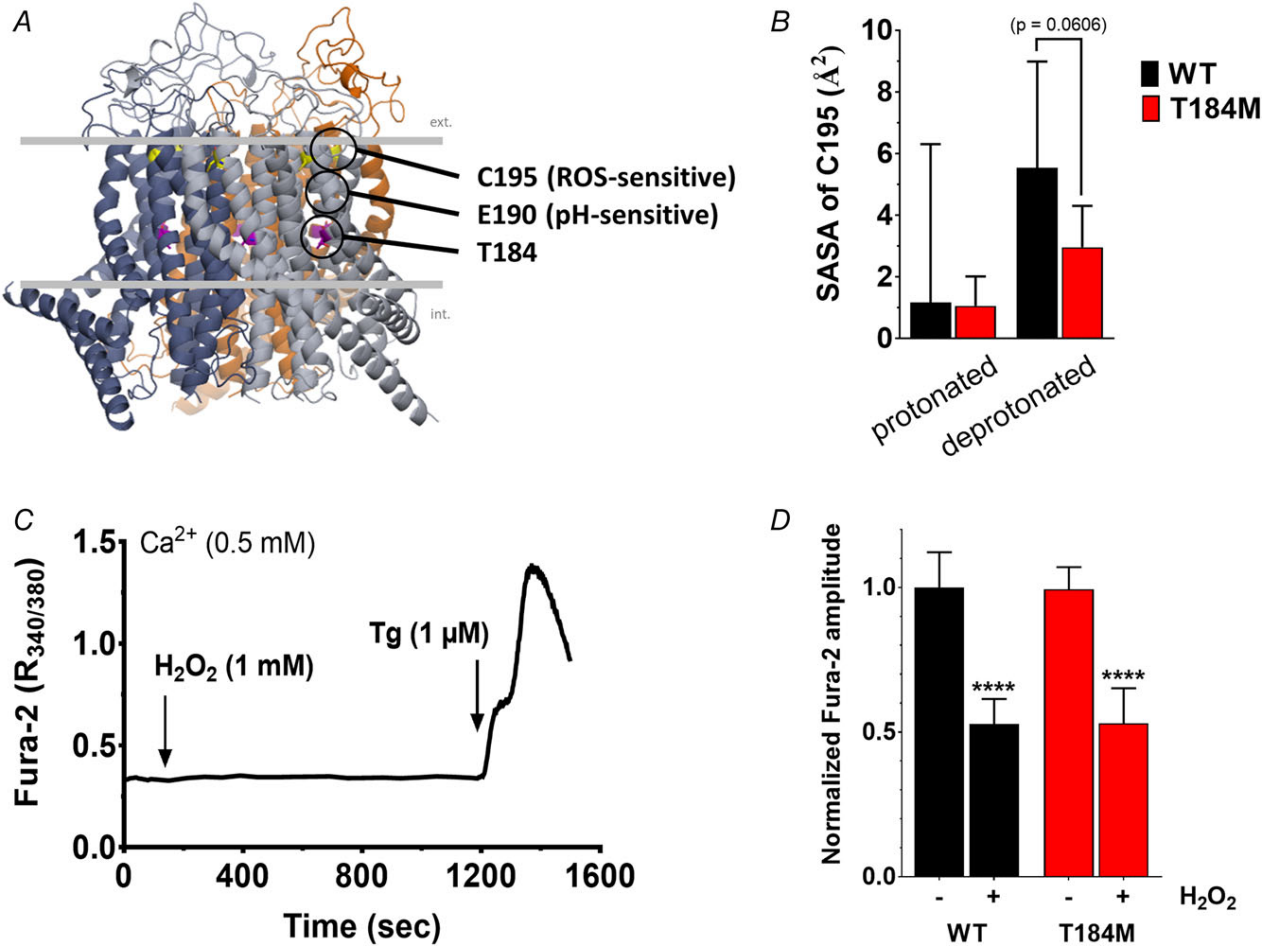
ORAI1‐T184M is sensitive to H_2_O_2_ inhibition *A*, structural model of human ORAI1 showing the reactive cysteine C195 and the pH‐sensing E190 residue close to the T184M mutation. *B*, solvent accessible surface area (SASA) of C195 in ORAI1‐WT and ‐T184M, with E190 in the protonated or deprotonated state. Data are mean ± 95% CI of 5 (deprotonated) or 2 (protonated) simulations (two‐tailed Kruskal–Wallis test). *C*, HEK‐293T cells co‐expressing STIM1 and ORAI1‐WT or ‐T184M were treated with 1 mm H_2_O_2_ for 18 min in the presence of 0.5 mm Ca^2+^, then exposed to 1 μm Tg to evoke SOCE. *D*, statistical evaluation of the Ca^2+^ elevations evoked by Tg in control and H_2_O_2_ treated cells. Data are means ± 95% CI of 24–33 cells from 4 independent experiments. A two‐tailed unpaired Student's *t* test with Welsh's correction was used to assess significant differences between WT and T184M or between treated and untreated cells within each group.

Reactive oxygen species (ROS) negatively modulate ORAI1 function via the reversible oxidation of reactive cysteine residues in the second and third TM domains (Bogeski *et al*. 2010; Alansary *et al*. 2016). Replacement of T184 by a bulky methionine could affect the helix conformation and hinder the accessibility of the reactive cysteine in TM3 (C195) to extracellular ROS. To investigate this possibility, we performed molecular dynamics simulations (MDSs) to evaluate the solvent accessible surface area (SASA) of the C195 residue in the WT and T184M backgrounds (Fig. 5 *A* and *B* and Supporting information videos S1–S4). The SASA of the C195 residue was not altered by the T184M mutation when the simulation was ran with the TM3 in the protonated state and was appreciably, but not significantly lower in the T184M than in the WT channel in the deprotonated state of E190 (Fig. 5 *B*). This indicates that the accessibility of the reactive cysteine at position 195 depends on the protonated state of the ORAI channel and might be restricted by the T184M mutation at alkaline pH. To directly test the accessibility of C195 to oxidants, we monitored SOCE in cells exposed to 1 mm H_2_O_2_ prior to store depletion (Fig. 5 *C*). Pretreatment with H_2_O_2_ reduced SOCE amplitude by half, regardless of whether ORAI1‐WT or ‐T184M was expressed (Fig. 5 *D*). These data indicate that the sensitivity of ORAI1‐T184M to external ROS is conserved and are consistent with the MDSs since the E190 residue is likely to be protonated at physiological pH.

### ORAI1‐V107M and ‐T184M are gated more efficiently by STIM1

**Figure 6:**
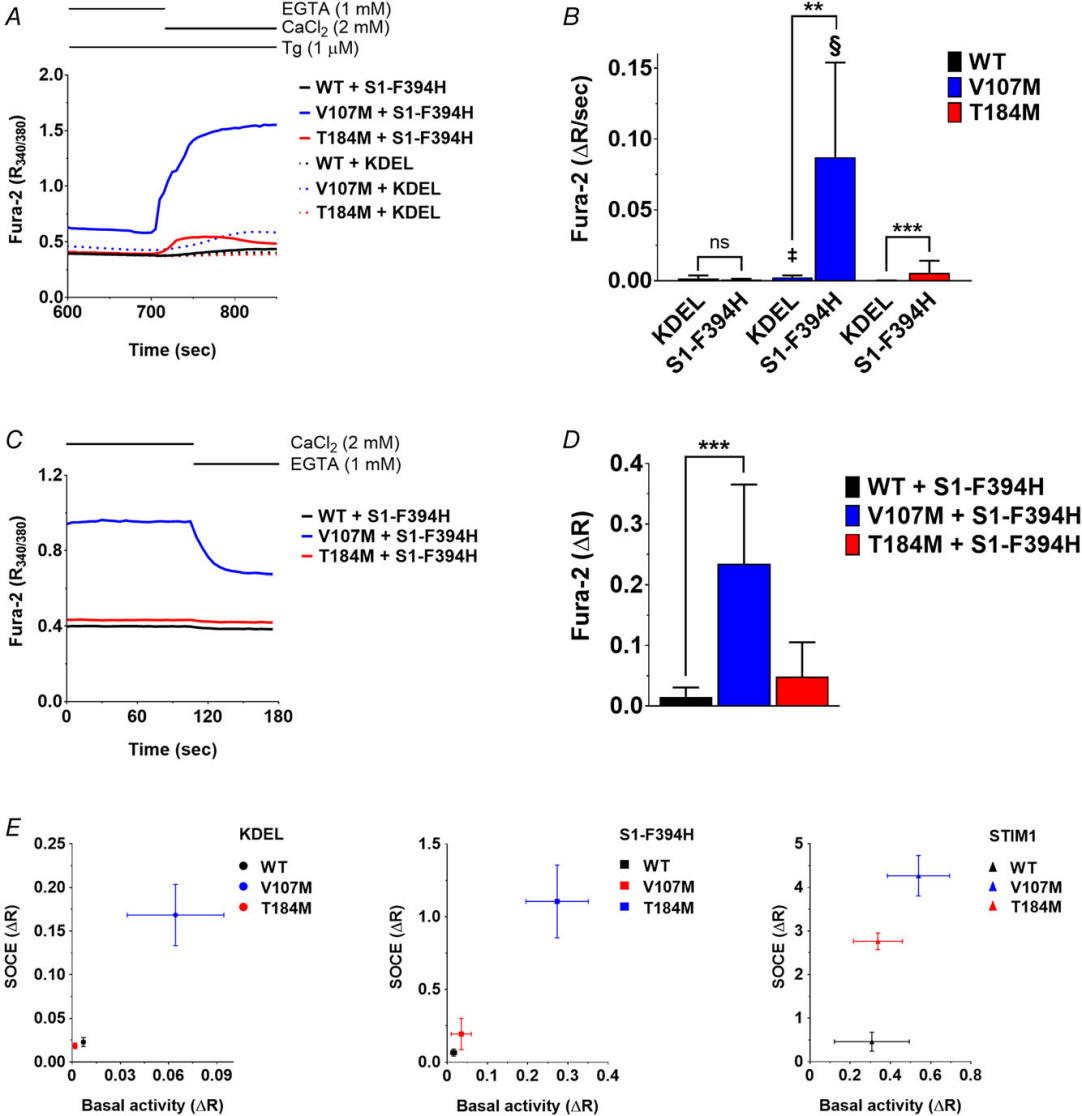
The gating‐deficient STIM1‐F394H activates ORAI1‐V107M and ‐T184M *A*, mean responses evoked by readmitting Ca^2+^ to Tg‐treated *Stim1^−/−^/Stim2^−/−^* (DKO) mouse embryonic fibroblasts co‐expressing the binding‐deficient STIM1‐F394H together with ORAI1‐WT, ‐V107M or ‐T184M. *B*, statistical evaluations of the slope of the response shown in *A* and of control responses recorded in DKO cells co‐expressing TagRFP‐KDEL (KDEL) with the ORAI1 constructs. Data are means ± 95% CI of 15–43 cells. The two‐tailed Mann–Whitney and Kruskal–Wallis statistical tests were used to assess significant differences between KDEL and STIM1‐F394H within each group or between the WT and TAM‐related ORAI1 channels respectively (§*P* ≤ 0.0001, ‡*P* ≤ 0.001). *C*, mean responses evoked by Ca^2+^ removal in DKO cells expressing STIM1‐F394H and the ORAI1 constructs prior to store depletion. *D*, statistical evaluations of the amplitude of the responses in *C*, reporting basal Ca^2+^ entry. Data are means ± 95% CI of 19–22 cells (two‐tailed Kruskal–Wallis test). *E*, SOCE amplitude *vs*. basal Ca^2+^ entry in DKO cells co‐expressing ORAI1‐WT, ‐V107M or ‐T184M together with TagRFP‐KDEL (left), STIM1‐F394H (middle) or STIM1 (right). Data are means ± SEM of 12–42 cells from 2 independent experiments.

We previously reported that the constitutive activity of ORAI1‐T184M is absent in *Stim1^−/−^/Stim2^−/−^* (DKO) mouse embryonic fibroblasts, implying a strict requirement for STIM proteins for channel gating (Bohm *et al*. 2017). This suggests that the T184M mutation enhances the binding affinity of ORAI1 for endogenous STIM proteins, thereby facilitating the transmission of the gating signal in the absence of store depletion. To test this hypothesis, we took advantage of a gating‐deficient STIM1 mutant (STIM1‐F394H) reportedly unable to cluster and gate ORAI1 channels unless low concentrations of 2‐aminoethyl diphenylborinate (2‐APB) are added to enhance STIM1–ORAI1 coupling (Zhou *et al*. 2015). STIM1‐F394H was overexpressed in DKO cells together with WT ORAI1 or the TAM‐associated ORAI1 mutants, and its ability to restore SOCE was compared to that of WT STIM1 or an ER‐targeted TagRFP (KDEL), used as a negative control. As expected, SOCE was absent in DKO cells expressing the control KDEL together with ORAI1‐WT or ‐T184M and was detectable in cells expressing KDEL and ORAI1‐V107M, reflecting the STIM1‐independent constitutive activity of the V107M channel (Fig. 6 *A*, *B* and *E* left panel). SOCE remained abrogated in DKO cells co‐expressing STIM1‐F394H and ORAI1‐WT, validating the loss of activity of the gating‐deficient STIM1 mutant. However, STIM1‐F394H mediated significant SOCE when co‐expressed with ORAI1‐T184M and strongly enhanced the activity of V107M‐ORAI1 compared to the KDEL control condition, indicating that the two TAM‐associated channels can be activated by this STIM1 mutant (Fig. 6 *A*, *B* and *E* middle panel). Constitutive Ca^2+^ entry, estimated from responses evoked by Ca^2+^ removal before store depletion, was significant in cells expressing STIM1‐F394H together with ORAI1‐V107M, detectable with ORAI1‐T184M (without reaching statistical significance) and absent with ORAI1‐WT (Fig. 6 *C* and *D*). The basal Ca^2+^ fluxes were 5–6 times lower than those measured after store depletion (Fig. 6 *E* middle panel), suggesting that the coupling between STIM1‐F394H and the TAM‐associated ORAI1 mutants was enhanced by store depletion. These data confirm that the ORAI1‐T184M channel has an increased affinity for STIM1, explaining the gain‐of‐function occurring at endogenous STIM1 levels in skeletal muscle cells. Mutation V107M is overactive in the absence of STIM1, and its activity is further potentiated by the addition of WT or binding‐deficient STIM1 (Fig. 6 *E* middle and right panels).

**Figure 7:**
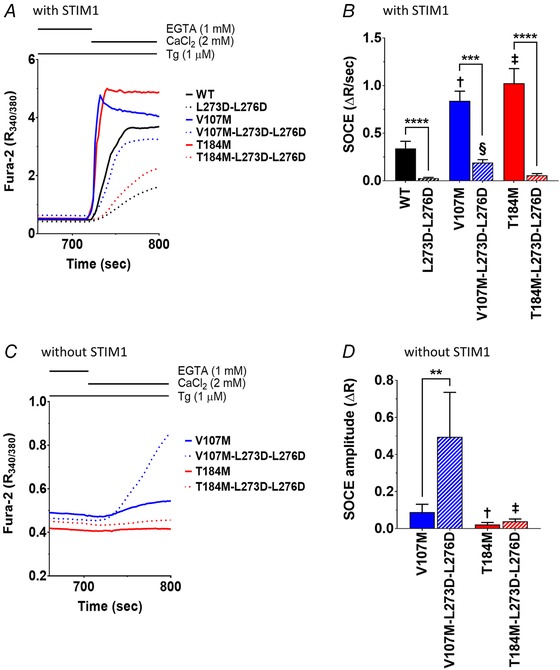
The L273D–L276D mutation decrease TAM channels store‐operated activity and increase ORAI1‐V107M constitutive activity *A*, mean SOCE responses in DKO mouse embryonic fibroblasts co‐expressing STIM1 together with ORAI1‐WT, ‐V107M, ‐T184M, with or without the additional mutations L273D–L276D in the ORAI1 C‐terminus. *B*, statistical evaluations of the slope of the responses shown in *A*. Data are means ± 95% CI of 21–53 cells from 5 independent experiments (two‐tailed Kruskal–Wallis test, §*P* ≤ 0.0001, ‡*P* ≤ 0.001, †*P* ≤ 0.01). *C*, mean responses evoked by readmitting Ca^2+^ to Tg‐treated DKO cells expressing ORAI1‐V107M or ‐T184M, with or without the additional C‐terminal mutations L273D–L276D, in the absence of STIM. *D*, statistical evaluation of the responses illustrated in *C*. Data are means ± 95% CI of 12–47 cells from 5 independent experiments (two‐tailed unpaired *t* test with Welsh's correction, ‡*P* ≤ 0.001, †*P* ≤ 0.01).

To further confirm that ORAI1‐T184M gain‐of‐function relies on STIM1‐mediated gating, we mutated two leucine residues at position 273 and 276 on the ORAI1 C‐terminus reportedly mediating the interaction between the channel and STIM1 (Calloway *et al*. 2010; Tirado‐Lee *et al*. 2015). The L273D–L276D mutation decreased SOCE by 90% in WT and T184M channels and by 75% in V107M channels co‐expressed with STIM1 in DKO cells (Fig. 7 *A* and *B*). This confirms that the double leucine mutation reduces the affinity of the ORAI1 channel for STIM1 and indicates that the gain‐of‐function of the V107M, but not of the T184M channel, persists in the presence of a disrupted STIM1 binding site. We then assessed the constitutive activity of these triple mutant channel. The L273D–L276D mutation had no effect on the T184M channel in the absence of STIM1, but unexpectedly increased constitutive ORAI1‐V107M Ca^2+^ fluxes by ∼5‐fold (Fig. 7 *C* and *D*). These data indicate that mutations in either the TM1 or the TM3 domain of ORAI1 enhance the gating probability of the channel by increasing its coupling efficiency to STIM1. The ORAI1‐T184M gain‐of‐function is strictly dependent on STIM1 binding while a disrupted STIM1/ORAI1 interface impacts the constitutive activity of the ORAI1‐V107M channel.

### Structural effects of ORAI1‐V107M and ‐T184M mutations on the channel pore

**Figure 8:**
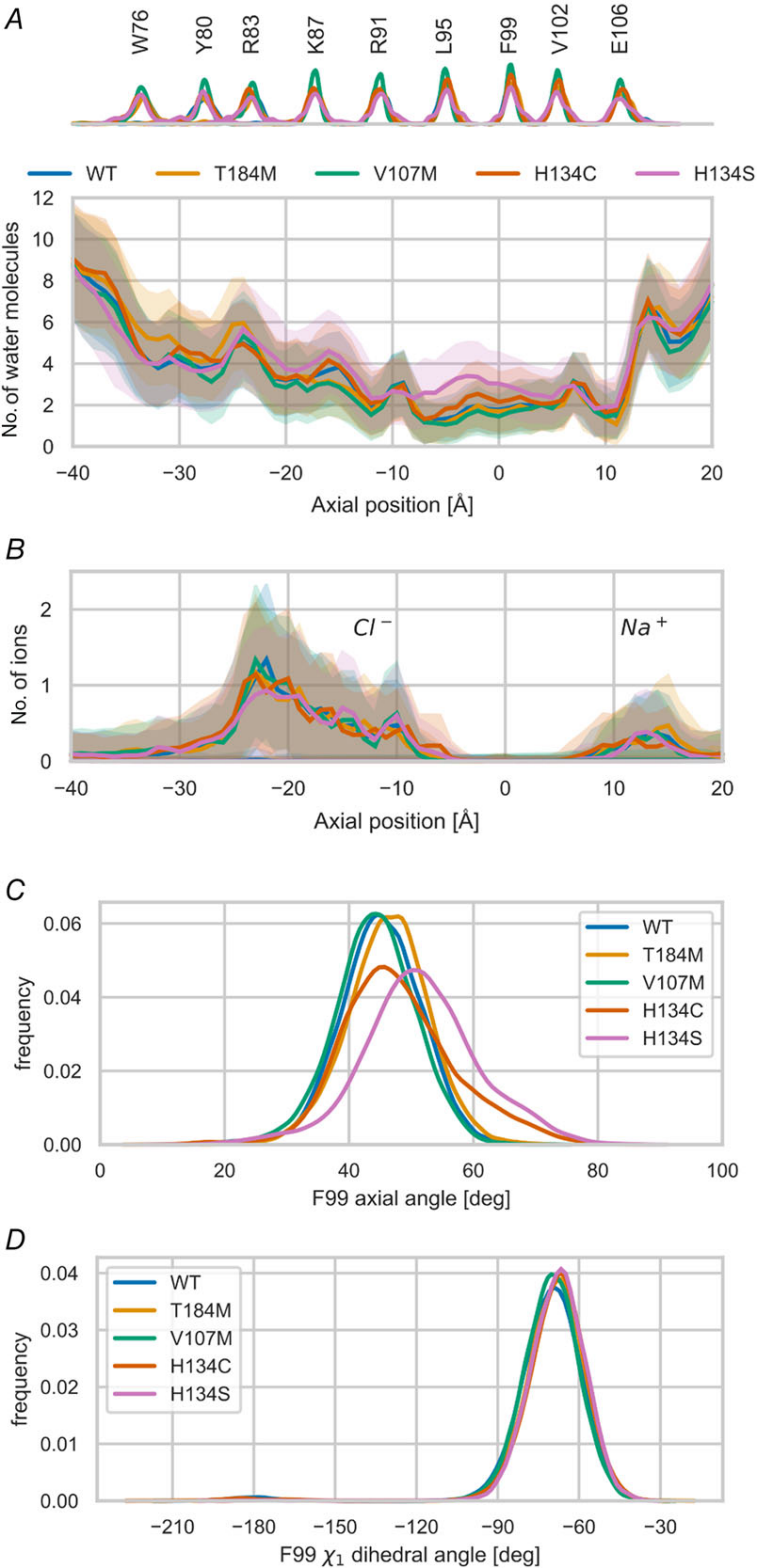
Molecular dynamics simulations of ORAI1‐V107M and ‐T184M pore permeability *A*, water occupancy in a 10 Å radius cylinder along the channel pore for the TAM ORAI1 mutants and the constitutively open channels H134C and H134S. The distribution of C_α_ atoms of various residues from TM1 along the pore axis is shown on the top of the panel. *B*, penetration of sodium (Na^+^) and chloride (Cl^−^) ions in a 10 Å cylinder along the channel pore for the various mutants. *C*, distribution of the axial angle of the F99 side‐chain as described in Yeung *et al*. (2018) over the course of MD trajectories. *D*, distribution of the χ_1_ dihedral angle of the F99 side‐chain, as defined by atoms N, C_α_, C_β_, C_γ1_, over the course of the MD trajectories.

To assess the structural effects of the V107M and T184M mutations on the residues facing the channel pore, we performed MDSs based on the closed conformation of our hORAI1 model, using two ORAI1 mutants previously shown to be constitutively open, H134C and H134S (Frischauf *et al*. 2017; Yeung *et al*. 2018), as positive controls. ORAI1‐H134S, and to a lesser extent ‐H134C, showed higher protein solvation along the pore, similar to Yeung *et al*. (2018), but no change in water penetration was detected for the V107M or T184M mutants compared to the WT channel (Fig. 8 *A*). Rotation of the F99 side‐chain relative to the pore axis has been proposed to render the pore of H134 mutants permeable to cations. Our simulations reported a discrete rotation of the F99 axial for ORAI1‐H134C, but a conserved pore axis conformation for the T184M and V107M mutants (Fig. 8 *C*). Penetration of Na^+^ and Cl^−^ ions into the channel pore was also conserved (Fig. 8 *C*), as was the χ_1_ dihedral angle of the F99 side‐chain (Fig. 8 *D*). Therefore, our MDSs suggest that mutations V107M and T184M do not directly alter the orientation of the residues facing the channel pore of the ORAI1 channel, under its closed conformation.

## Discussion

The ubiquitous SOCE mechanism mediated by STIM and ORAI proteins has distinctive characteristics in skeletal muscle. SOCE activates within milliseconds in skeletal muscle cells, while the sequence of events linking store depletion to ORAI1 gating typically takes several tens of seconds in other tissues (Launikonis & Rios, 2007; Edwards *et al*. 2010; Koenig *et al*. 2018). The peculiar architecture of the SR permanently facing the PM might account for the rapid SOCE activation as might the preferential expression of the longer STIM1L splice variant in skeletal muscle (Darbellay *et al*. 2011). The SR is also more resilient to Ca^2+^ depletion due to its high Ca^2+^ buffering capacity and the large amount of SERCA pumps (Payne *et al*. 2009), and whether the SR Ca^2+^ content decreases sufficiently during physiological muscle activity to stimulate SOCE has been challenged (Cully & Launikonis, 2013). A recent study reported that SOCE is activated after every action potential in skinned fast twitch rat muscle fibres, suggesting that SOCE acts as a counter‐flux to T‐system Ca^2+^ extrusion to maintain Ca^2+^ homeostasis during muscle contraction (Koenig *et al*. 2018). Several studies reported an increased muscle fatigue in the absence of SOCE, linked to faster decline in Ca^2+^ content upon repetitive membrane depolarizations (Stiber *et al*. 2008; Wei‐Lapierre *et al*. 2013; Sztretye *et al*. 2017), but unexpectedly Orai1‐deficient mice had no endurance deficit (Carrell *et al*. 2016).

SOCE is clearly important for skeletal muscle function in humans, because gain‐of‐function mutations in the *ORAI1* gene are causally related to TAM, a muscular disease with elevated blood CK levels and loss of fast twitch (type II) fibres caused by Ca^2+^ overload (Rosenberg *et al*. 1985; Bohm *et al*. 2013). TAM patients also suffer from prolonged contractions during effort, consistent with a role of ORAI1 and SOCE in the maintenance of human muscle Ca^2+^ balance. Human ORAI1 gain‐of‐function mutations therefore highlight the reliance of skeletal muscle on SOCE and provide valuable insights into the molecular basis of the permeation and gating mechanism of ORAI channels. Here, we characterized the biophysical, regulatory and pharmacological properties of two TAM‐associated mutations, one located in the outer vestibule of the channel pore (V107M) and another in the middle of the third transmembrane domain (T184M). Using electrophysiology and Ca^2+^ imaging, we establish that the V107M mutation decreases the Ca^2+^ selectivity and pH sensitivity of the ORAI1 channel. In contrast, the Ca^2+^ selectivity, pH sensitivity and ROS sensitivity of ORAI1 are not affected by the T184M mutation. Both mutations increase the efficiency of the STIM1 gating signal, leading to a gain‐of‐function phenotype that, in the case of the T184M mutation, is strictly dependent on STIM1.

In this study, we confirm that ORAI1‐V107M and ‐T184M channels are constitutively active and mediate enhanced SOCE upon store depletion in HEK‐293T cells, a gain‐of‐function phenotype that we previously reported to be independent of the formation of channel clusters (Bohm *et al*. 2017). We extend these findings by showing that the basal activity of these two TAM‐associated channels is also detected in human primary myoblasts, and that both retain sensitivity to GSK‐7975A, a selective inhibitor of ORAI1 and ORAI3 efficient in models of acute pancreatitis (Derler *et al*. 2008; Gerasimenko *et al*. 2013). GSK‐7975A inhibits ORAI1‐mediated CRAC currents without altering STIM1 oligomerization or STIM1–ORAI1 interactions (Derler *et al*. 2013) and, in our hands, completely abolished Ca^2+^ fluxes mediated by ORAI1‐V107M or ‐T184M at micromolar concentrations in both naive and store‐depleted cells. In addition, the inhibitor efficiently blocked the constitutive influx of two other TAM‐associated ORAI1 mutants, L138F and P245L, but not of the G98S pore mutant, validating the therapeutic use of the compound in some but not all cases of TAM. Interestingly, half‐maximal inhibition was achieved at lower GSK‐7975A concentrations for mutations in the TM4 and TM2–TM3 outer rings than for mutations in the central TM1, suggesting that the compound is more efficient on channels with conserved pore residues.

The high Ca^2+^ selectivity of the ORAI1 channel is conferred by a ring of negatively charged glutamate residues near the extracellular face of the channel pore. Amino acid substitutions of this selectivity filter (E106) or in the nearby TM1–TM2 loop (D110, D112, D114) dramatically alter channel selectivity to allow permeation of monovalent cations (Prakriya *et al*. 2006; Vig *et al*. 2006a; McNally *et al*. 2009; Frischauf *et al*. 2015). Our ion substitution experiments establish that the TAM‐associated mutation V107M vicinal to the selectivity filter alters channel selectivity, causing substantial sodium permeation across the ORAI1‐V107M channel. We speculate that replacement of V107 by the bulky methionine side‐chain disrupts the adjacent E106 ring that binds incoming Ca^2+^ ions, causing the selectivity defect observed in the mutated channel. Our MDSs, however, failed to reveal any alteration of the channel pore permeation for water molecules or Na^+^ ions. These simulations were based on the structure of the *Drosophila* Orai1 channel in its closed conformation, a factor that might have impeded the detection of the impact of the V107M mutation on the channel selectivity filter. Interestingly, we observed that the V107M TAM mutant regained Ca^2+^ selectivity when co‐expressed with STIM1, as reported for the V102C synthetic mutant (McNally *et al*. 2012). MDSs of the ORAI1 pore helix in the resting and open conformation suggest that STIM1 binding induces a rotation of the TM1 domain (Yeung *et al*. 2017, 2018). However, we could not detect alterations in the conformational changes in our MDSs of the TAM‐associated channel mutants, and thus whether a rotation of critical TM1 residues underlies the STIM1‐mediated gain in Ca^2+^ selectivity of the ORAI1‐V107 channel remains to be established.

Acidic solutions reduce Ca^2+^ currents mediated by ORAI1 channels, a regulatory mechanism that might protect skeletal muscle fibres from Ca^2+^ overload as the external pH decreases during exercise (Matsuda *et al*. 1995). The pH sensitivity of ORAI is reportedly mediated by a residue in TM3 (E190) located near the T184M TAM mutation (Beck *et al*. 2014; Tsujikawa *et al*. 2015). Contrary to our expectation, inhibition by acidic pH was preserved in the T184M mutant and reduced in the V107M mutant, suggesting that mutations in TM1 rather than in TM3 alter the accessibility of the pH sensing residue(s). The reduced pH sensitivity of ORAI1‐V107M might exacerbate the deleterious effects of this overactive channel in skeletal muscle cells of affected patients, explaining the aggravation of the muscle contractures and the increased fatigability observed during effort.

Besides pH, the ORAI1 channel is negatively modulated by ROS, a mechanism that might also protect muscle fibres from persistent Ca^2+^ influx as the oxidative state of the muscle tissue increases during repeated muscular contractions (Reid *et al*. 1992; Powers *et al*. 2011). We surmised that the T184M mutation in TM3 might render the channel resistant to ROS‐mediated inhibition by reducing the solvent accessibility of the nearby cysteine at position 195, which was shown to be reversibly oxidized by H_2_O_2_ (Bogeski *et al*. 2010; Alansary *et al*. 2016). Our MDSs indicate that solvent accessibility might indeed differ but only at alkaline pH, and our Ca^2+^ imaging experiments establish that the mutated channel retains normal sensitivity to H_2_O_2_ in physiological saline buffer. Inhibition by ROS is consistent with the absence of muscular symptoms associated with exercise in the patient bearing the *ORAI1* T184M mutation (Bohm *et al*. 2017), yet the presence of sarcoplasmic aggregates in muscle biopsies indicate that his muscle fibres experienced sustained Ca^2+^ overload. Our Ca^2+^ imaging and electrophysiological results confirm that T184M is indeed a gain‐of‐function mutation and reveal that this particular mutation does not alter the Ca^2+^ selectivity of the ORAI1 channel or its inhibition by acidic pH and by ROS.

Remarkably, the gain‐of‐function conferred by the T184M mutation is strictly dependent on STIM. We previously showed that the constitutive activity of this channel is absent in cells lacking endogenous STIM proteins (Bohm *et al*. 2017). Our MDSs confirm that the T184M mutation does not alter water and ion permeation in the pore of the closed channel, and we now show that T184M‐mediated currents require STIM1 co‐expression and a functional interaction of the ORAI1 C‐terminus with STIM1. We further extend these findings by showing that Ca^2+^ entry through ORAI1‐T184M can be induced by the STIM1‐F394H mutant unable to gate WT‐ORAI1 channels, and that the gain‐of‐function is prevented by the L273D–L276D mutation that reduces STIM1 binding. This suggests that the T184M mutation renders the channel hypersensitive to potential ligands, likely by facilitating the conformational changes occurring during the activation step. The V107M mutant was also activated by STIM1‐F394H, but the gain‐of‐function persisted with the L273D–L276D mutation, which unexpectedly increased the constitutive activity of the V107M channel. These data indicate that both mutations increase ORAI1 coupling efficiency to STIM1, and indicate that a disrupted STIM1/ORAI1 interface alters the constitutive, STIM1‐independent activity of the ORAI1‐V107M channel. STIM1–ORAI1 coupling was proposed to involve a gating signal initiated at TM4, relayed via TM3 and TM2 into a rotation of TM1 causing channel opening (Yeung *et al*. 2017). The methionine substitution at position 184 might induce a kink in the TM3 helix that could facilitate the transmission of the signal from TM4 to TM2. The V107M mutation, on the other hand, renders the channel leaky and thus disrupts the TM1 pore already in the closed state, and the conductance of the leaky V107M channel can be further enhanced by the gating‐deficient STIM1‐F394H mutant. This mutation also disrupts the Ca^2+^ selectivity filter to favour the permeation of monovalent cations, an effect that is alleviated by STIM1 binding. We propose that the V107M channel responds more readily to a rotation of the TM1 hydrophobic residues and that the conformational change occurring during channel opening restores a ring of glutamate residues at the pore entrance, allowing the mutated channel to regain Ca^2+^ selectivity. The endogenous ligand activating the mutated channels in skeletal muscle cells remains to be identified. STIM2 is a good candidate as it has a higher lipid‐binding affinity (Bhardwaj *et al*. 2013) and responds to mild Ca^2+^ depletion of the ER (Brandman *et al*. 2007; Parvez *et al*. 2008), but STIM1L should also be investigated, as it is recruited to cortical ER by the Ca^2+^ depletion due to the contractile activity of muscle cells (Darbellay *et al*. 2011; Sauc *et al*. 2015).

In conclusion, our study establishes the molecular mechanisms underlying the gain‐of‐function conferred by the V107M and T184M mutations leading to TAM. Mutation V107M vicinal to the selectivity filter increases ORAI1 channel permeability to cations and decreases its Ca^2+^ selectivity in a STIM1‐dependent manner, likely by destabilizing the pore helix. It also reduces channel inhibition at acidic pH, potentially accounting for the aggravation of muscular symptoms during exercise in affected patients. The gain‐of‐function of the T184M mutation in the outer TM3 ring is only revealed by the presence of STIM proteins. T184M does not alter ORAI1 Ca^2+^ selectivity or its inhibition by acidic pH and ROS but renders the channel hypersensitive to intracellular ligands, likely by facilitating the transmission of the gating signal initiated at the channel's C‐terminal tail. Both channel mutants retain sensitivity to GSK‐7975A, providing a potential therapeutic strategy to treat the muscular symptoms associated with TAM.

## Additional information

### Competing interests

None of the author reports conflicts of interest.

### Author contributions

N.D. designed and coordinated the study. N.D. and M.B. wrote the manuscript. M.B. performed the Ca^2+^ imaging and electrophysiology experiments, under the supervision of M.F. The effect of GSK‐7975A on TAM‐associated mutants was assessed by R.B. and J.H.K. Molecular dynamics simulations were done by G.G. All authors designated are qualified for authorship. All authors have read and approved the final version of this manuscript and agree to be accountable for all aspects of the work in ensuring that questions related to the accuracy or integrity of any part of the work are appropriately investigated and resolved. All persons designated as authors qualify for authorship, and all those who qualify for authorship are listed.

### Funding

This work was supported by Swiss National Foundation (SNF) grants 31003A‐149566 (to N.D.), 323530‐158118 (to M.B.), and 310030‐166313 (to M.F.), the Sinergia SNF grant CRSII3‐16078 (to N.D. and M.A.H.), and the Korean‐Swiss Science and Technology Programme EG 05‐122016 (to J.H.K.).

## Supporting information


**Video S1. hORAI1 WT, E190 deprotonated**

**Video S2. hORAI1 T184M, E190 deprotonated**

**Video S3. hORAI1 WT, E190 protonated**

**Video S4. hORAI1 T184M, E190 protonated**

Videos showing protein and ion movement during the trajectories of selected representative simulations under four conditions. In the videos, the hORAI1 hexamer is shown in cartoon representation with each monomer coloured differently, with the two monomers closest to the camera not shown for clarity. Lipid molecules of the membrane bilayer are also hidden for clarity. Yellow and cyan spheres represent sodium and chloride ions, respectively. Water molecules are shown in a red‐white stick representation. The videos were generated using the Visual Molecular Dynamics (VMD) software (Humphrey *et al*. 1996).
